# Substrate-Favored Lysosomal and Proteasomal Pathways Participate in the Normal Balance Control of Insulin Precursor Maturation and Disposal in β-Cells

**DOI:** 10.1371/journal.pone.0027647

**Published:** 2011-11-10

**Authors:** Xiaoping Zhang, Qingxin Yuan, Wei Tang, Jingyu Gu, Kwame Osei, Jie Wang

**Affiliations:** Division of Endocrinology, Diabetes and Metabolism, Department of Internal Medicine, The Ohio State University, Columbus, Ohio, United States of America; Consejo Superior de Investigaciones Cientificas, Spain

## Abstract

Our recent studies have uncovered that aggregation-prone proinsulin preserves a low relative folding rate and maintains a homeostatic balance of natively and non-natively folded states (i.e., proinsulin homeostasis, PIHO) in β-cells as a result of the integration of maturation and disposal processes. Control of precursor maturation and disposal is thus an early regulative mechanism in the insulin production of β-cells. Herein, we show pathways involved in the disposal of endogenous proinsulin at the early secretory pathway. We conducted metabolic-labeling, immunoblotting, and immunohistochemistry studies to examine the effects of selective proteasome and lysosome or autophagy inhibitors on the kinetics of proinsulin and control proteins in various post-translational courses. Our metabolic-labeling studies found that the main lysosomal and ancillary proteasomal pathways participate in the heavy clearance of insulin precursor in mouse islets/β-cells cultured at the mimic physiological glucose concentrations. Further immunoblotting and immunohistochemistry studies in cloned β-cells validated that among secretory proteins, insulin precursor is heavily and preferentially removed. The rapid disposal of a large amount of insulin precursor after translation is achieved mainly through lysosomal autophagy and the subsequent basal disposals are carried out by both lysosomal and proteasomal pathways within a 30 to 60-minute post-translational process. The findings provide the first clear demonstration that lysosomal and proteasomal pathways both play roles in the normal maintenance of PIHO for insulin production, and defined the physiological participation of lysosomal autophagy in the protein quality control at the early secretory pathway of pancreatic β-cells.

## Introduction

Polypeptide homeostasis in normal cells (PHIC) is a dynamic equilibrium maintained by integral processes, such as the synthesis, folding, clearance, and transport of proteins to functional destinations [1]. Perturbations of PHIC can result in various diseases, including early-onset diabetes in the *Ins2^+/Akita^* mice and up to 20% of neonatal diabetes in humans [2]–[4], which result primarily from proinsulin homeostasis disorders induced by varied mutations in the preproinsulin molecule [1]. In eukaryotic cells, the lysosomal and proteasomal pathways are the two main routes responsible for protein disposal in the normal maintenance of PHIC. Proteasomes mainly clean misfolded proteins marked by (poly)ubiquitination [5], [6], and lysosomes digest cytosolic cargo through macroautophagy, microautophagy, and chaperone-mediated autophagy (CMA) pathways [7], [8]. Multiple clearance mechanisms are implicated in the homeostasis of individual proteins in cells. For example, the macroautophagy, CMA, and proteasomal pathways all participate in clearance of wild-type α-synuclein, a cytosolic protein associated with Parkinson disease [9], [10].

It is recognized that in eukaryotic cells about one-fifth to one-third of nascent proteins are targeted to the endoplasmic reticulum (ER) wherein structural maturation and modifications of secretory protein take place for transit to the intracompartmental lumen and membrane, and ultimate secretion [5], [6]. Normally, a set of molecular helpers, such as various chaperones and adaptors, assist maturation. Recent findings reveal that under stress, adaptive mechanisms function to protect the ER (see reviews: 5,6,11,12), including the (a) translational attenuation and enhanced transcription of selected chaperone genes, (b) retro-translocation of misfolded proteins for ER-associated degradation (ERAD) by the proteasome, (c) ER-phagy (ER consumption by autophagy) [8], [13], [14], and (d) substrate-specific translocational attenuation [15] and proteasomal degradation of the proteins rejected by the ER translocon [16].

Under most physiological conditions, proteasomes can rapidly degrade approximately 30% of nascent proteins [17], which may include a fraction of proteins destined naturally for the secretory pathway. However, it remains unclear whether the proteasomal pathway alone or in combination with the lysosomal pathway participates in the normal post-translational disposal process of secretory proteins at the early secretory pathway. Although adaptive responses, such as ER-phagy, have been elucidated under stress conditions, the physiological significance of the lysosomal pathway in the early secretory pathway remains to be defined.

Pancreatic β-cells (hereafter termed “β-cells”) are equipped with a well developed secretory pathway for the production of insulin. Proinsulin is the most abundant insulin precursor made in the pancreatic β-cells [18]. Higher glucose stimulation can increase proinsulin synthesis 50 times at the translational level [19]. Proinsulin will be processed into insulin in a general 2-hour course [18] that involves an early complex process of folding/maturation mainly in the ER and Golgi compartments. After a general 40–60 min trip through the ER and Golgi, proinsulin moves into the *trans* Golgi compartment and/or immature granules wherein C-peptide is removed. As a result, proinsulin is converted to insulin and stored in mature granules ready for release [18]. Our recent studies have uncovered that proinsulin inherent with an aggregation-prone nature preserves a low relative folding rate compared to control secretory proteins [1]. Proinsulin thus maintains a homeostatic balance of natively and non-natively folded states (i.e., proinsulin homeostasis, PIHO) at the early secretory pathways of β-cells as a result of the integration of maturation and clearance processes [1]. However, it remains unclear whether the two main protein disposal pathways participate in the normal control of insulin precursor disposal in pancreatic β-cells.

In this study, we have taken advantage of the recently improved metabolic-labeling and C-peptide immunoblotting approaches [1] combined with immunohistochemistry studies and characterized the roles of proteasomes and lysosomes in the early post-translational processing of insulin precursor. Mouse islets/β-cells during 24-hour pre-experimental and various experimental periods were incubated in routinely applied culture media containing mimic physiological glucose concentrations (5.5 mM, cloned β-cells; 11 mM, islets). By examining the changes in levels/states of (un)labeled proinsulin and control proteins in various post-translational courses with selective proteasomal and/or autophagy lysosomal inhibition, we found that the lysosomal and proteasomal pathways both function in disposals of insulin precursor for the normal maintenance of PIHO during insulin production. We also define a physiological role of lysosomal autophagy in the customary quality control at the early secretory pathway of pancreatic β-cells for the first time.

## Results

### Lysosomal or proteasomal inhibition preserves ^35^S-labeled proinsulin

**Figure 1:**
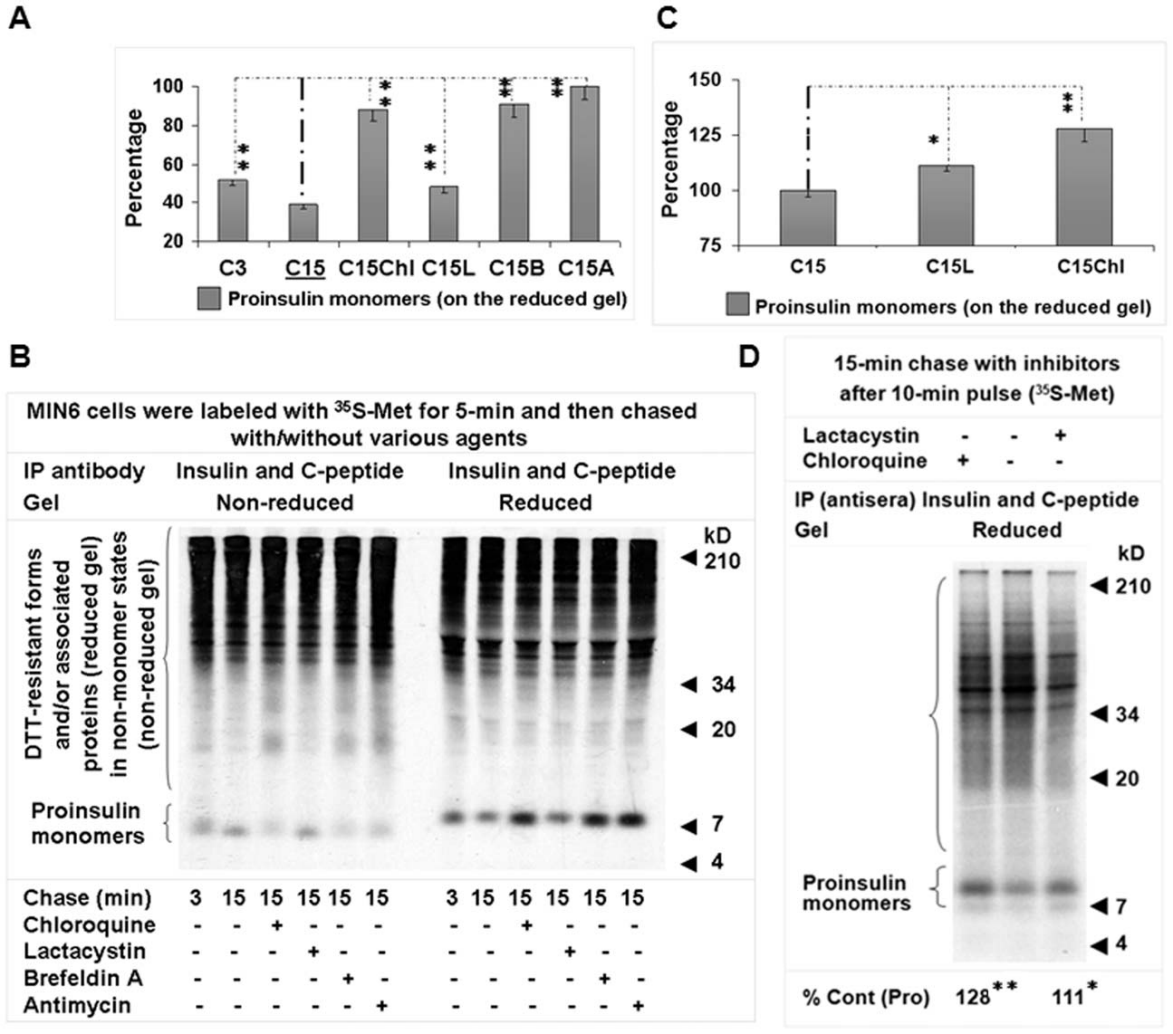
Lysosomal or proteasomal inhibition preserves ^35^S-methionine (Met) labeled nascent proinsulin in mouse islets/β-cells. After 24-hour pre-experimental culture with the 5.5 mM (MIN6 β-cells) or 11 mM (islets) glucose concentration, MIN6 β-cells (A and B) or islets (C) were chased for the indicated times with/without chloroquine (100 µg/mL), lactacystin (10 µM), antimycin (10 µM), or brefeldin A (10 µg/mL) after a pulse for the indicated times with ^35^S-Met. Cellular proteins were then subjected to immunoprecipitation with insulin and C-peptide antisera. Equal amounts of individual immunoprecipitates were resolved by 10% tricine non-reduced and/or reduced SDS-PAGE for radiography. Relative levels of ^35^S-proinsulin monomers in (B) or (D) of individual immunoprecipitates (on the reduced gel) were shown in (A) and (C), respectively. The data in (A) or (C) were reported as mean ± SD. n = 3. *P<0.05; **P<0.01. % cont (Pro): percentage of proinsulin monomer level compared to the control.

During a 15-minute chase, we compared the effect of lactacystin (10 µM) or chloroquine (100 µg/mL) with that of controls, e.g., antimycin (10 µM) or brefeldin A (10 µg/mL), on the kinetics of proinsulin labeled with ^35^S-methionine (Met) for 5 minutes in MIN6 β-cells. Met residue occurs only in proinsulin 2 in rodents, not in proinsulin 1. In this 15-minute chase, just as we previously observed [1], addition of the ATP production inhibitor antimycin largely blocked a heavy clearance of proinsulin, and a plateau of ^35^S-proinsulin appeared (refer to the monomers on the reduced gel). The proinsulin level at the natural 3-minute chase was about 51% of the plateau and at the 15-minute chase, about 39% (Figure 1A, B; Table S1; *P*<0.01; n = 3). Results consistently indicate that 49% (51% from 100%, at 3 minutes) and 12% (39% from 51%%, at 15 minutes) of ^35^S-proinsulin molecules were removed by rapid disposal, a process occurring immediately following translation [1], and a subsequent basal clearance during the time frame required for proinsulin maturation and departure from the ER as demonstrated previously [18], [20]. Compared to the 39% proinsulin level after the 15-minute natural chase, 47% was preserved with the addition of chloroquine, an agent for autophagy measurements that inhibits lysosomal acidification [21], [22], and 9% was preserved with addition of lactacystin, a specific proteasomal inhibitor (*P*<0.01; n = 3). In addition, no proinsulin was detected in the immunoprecipitates of control sera/immunoglobulin (Figure S1), further supporting that proinsulin preserved by selective lysosomal or proteasomal inhibition and evident in the immunoprecipitates of insulin and C-peptide antisera is specifically detected and mainly destined for natural clearance.

Interestingly, a significant fraction of proinsulin was also preserved by adding brefeldin A, an agent targeting guanosine-5′-triphosphate exchange factors and interfering with the formation and conveyance of protein transport vesicles (Figure 1A, B). The molecular targets of brefeldin A appear (in)directly involved in the process of proinsulin disposal in addition to its known role in cellular protein traffic. Block of the routine path from the ER to the Golgi transport alone cannot fully explain the entire preservation of proinsulin by brefeldin A. An enlarged subcompartment of the ER after addition of brefeldin A, the “brefeldin A body” defined in islet β-cells [23], may be linked to proinsulin removal somehow. Comparison of proinsulin states between non-reduced and reduced gels (Figure 1B) showed that the proinsulin preserved by the applied agents was concentrated mainly in the non-monomer states, which are primarily formed *in vivo* [1].

Addition of lactacystin or chloroquine during a 15-minute chase after a 10-minute pulse resulted in an increase of about 11 or 28% in the level of ^35^S-proinsulin in cultured mouse islets (Figure 1C, D; control versus lactacystin, 100.0 ± 3.3 versus 111.3 ± 5.1%, *P*<0.05; control versus chloroquine, 100.0 ± 3.3 versus 128.1 ± 7.2%, *P*<0.01; n = 3), indicating the likely involvement of the lysosomal and proteasomal pathways in removal of nascent proinsulin in islets and cloned β-cells. A potent difference in the effects of chloroquine between MIN6 β-cells and islets is most likely due to possible diversities in the machinery of clearance and maturation. As observed previously [1], in insulin and C-peptide immunoprecipitates (Figure 1B, D), unidentified molecular helpers are more abundant in MIN6 β-cells than in islets (irrespective of a possibly non-specific immunoglobulin-binding fraction, and they appear in the upper area of the reduced gel. See Figure S1). The helpers are components of proinsulin non-monomer states, which appear in the upper area of the non-reduced gel, and could function in proinsulin disposal, maturation, and even transport [1]. The difference may also have been affected by the glucose concentrations (5.5 mM, cloned β-cells; 11 mM, islets) we applied during the one day pre-experimental, 30 minutes Met starvation, and experimental period to mimic normal mice glycemic levels as used generally in the *in vitro* culture of islets/β-cells.

### Validation of the roles of lysosomes and proteasomes in proinsulin removal

To confirm the roles of lysosomes and proteasomes, we used C-peptide immunoblot and assessed the level changes of newly synthesized proinsulin during a 2-hour post-translational process in which *Ins2^+/+^* β-cells [24] were exposed to cycloheximide (Chx; 100 µg/mL, a protein synthesis inhibitor) with/without lysosomal or proteasomal inhibitors. Like the MIN6 β-cells, the *Ins2^+/+^* β-cells preserve the basic characteristics of natural pancreatic β-cell progenitors, such as the expression and secretion of insulin in response to glucose (unpublished data), proinsulin states [1], and ER stress responses [24]. A 2-hour course generally covers a complete post-translational process from the appearance of nascent proinsulin to the release of insulin [18].

**Figure 2:**
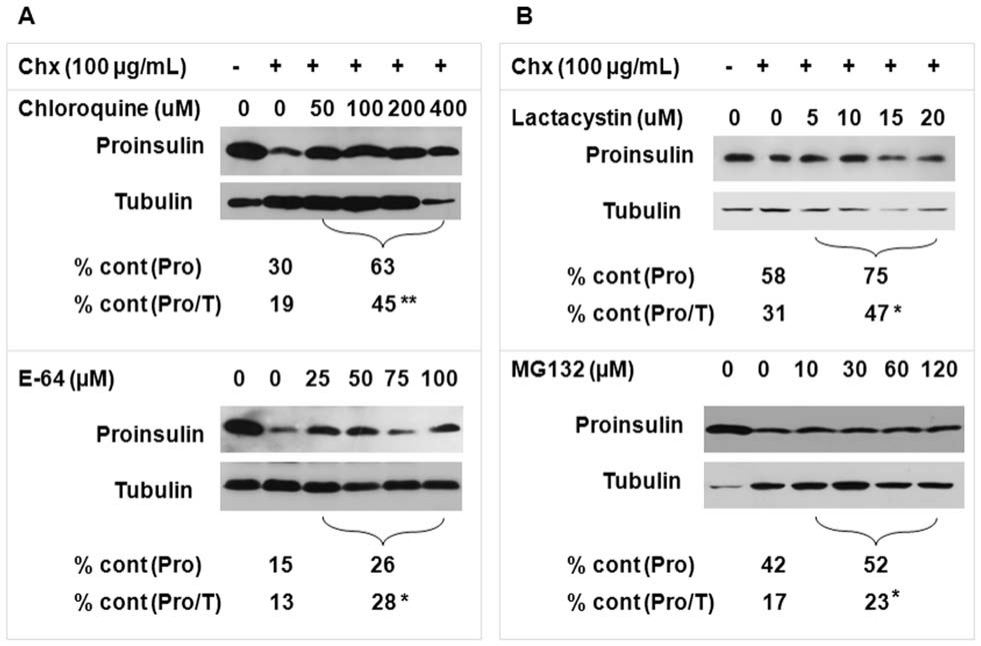
Validation of proteasomal or lysosomal roles in the disposal of proinsulin. After 24-hour pre-experimental culture with the 5.5 mM glucose concentration, the *Ins2^+/+^* β-cells were exposed to cycloheximide (Chx; 100 µg/mL); Chx and chloroquine (50–400 µg/mL); Chx and E-64 (25–100 µM); Chx and lactacystin (5–20 µM); or Chx and MG-132 (10–120 µM) for 2 hours with an untreated control. Cellular proteins (30 µg) were separated on16.5% tricine SDS-PAGE under reduced conditions and then examined by C-peptide or β-tubulin antibody on the same membrane. % cont (Pro): percentage of the (average) proinsulin level in individual treatments compared to the untreated control. % cont (Pro/T): percentage of the (average) proinsulin level in individual treatments compared to the untreated control and normalized by β-tubulin. The data were shown as mean. Statistical significances (*P<0.05; **P<0.01) of the normalized proinsulin level between Chx and individual treatments that included Chx were assessed by two-tailed t-test (n = 4).

Compared to the proinsulin level (normalized by β-tubulin if not specifically stated) in the untreated control, an average of 19% of proinsulin that was synthesized prior to the addition of the Chx was retained by adding Chx alone and 45.3%, by adding Chx and chloroquine (50–400 µg/mL, described as the Chx/chloroquine below for combined agents). Addition of trans-epoxysuccinyl-L-leucylamido-(4-guanidino)butane (E-64; 25–100 µM), a cysteine protease (e.g., cathepsins) inhibitor, caused retention of 27.8% of proinsulin versus 13.1% retained in the treatment with Chx alone (Figure 2, left panel; Chx versus Chx/Chlotoquine, 19.0 ± 5.1 versus 45.3 ± 8.2%, *P*<0.01; Chx versus Chx/E-64, 13.1 ± 5.3 versus 27.8 ± 6.1%, *P*<0.05; n = 4). The lower potency of E-64 than that of chloroquine may be partially attributable to the relatively limited targets of E64 versus the entire lysosomal lumen targeted by chloroquine. Compared to adding only Chx, addition of proteasomal inhibitor N-benzyloxycarbonyl-leucyl-leucyl-leucinal (MG-132; 10–20 µM) preserved 6% of proinsulin or addition of lactacystin (5–20 µM) preserved 16.4% (Figure 2, right panel; Chx versus Chx/MG-132, 17.1 ± 2.5 versus 23.1 ± 2.8%, *P*<0.05; Chx versus Chx/lactacystin, 31.0 ± 4.9 versus 47.4 ± 6.2%, *P*<0.05; n =  4). No significantly dose-dependent effect of individual inhibitors was observed despite a somewhat relevance between dose and effect appeared only in lower ranges of the lysosomal inhibitors. Both metabolic-labeling and immunoblotting analyses (Figures 1, 2) strongly suggest the participation of proteasomal and lysosomal pathways in endogenous proinsulin clearance in β-cells.

**Figure 3:**
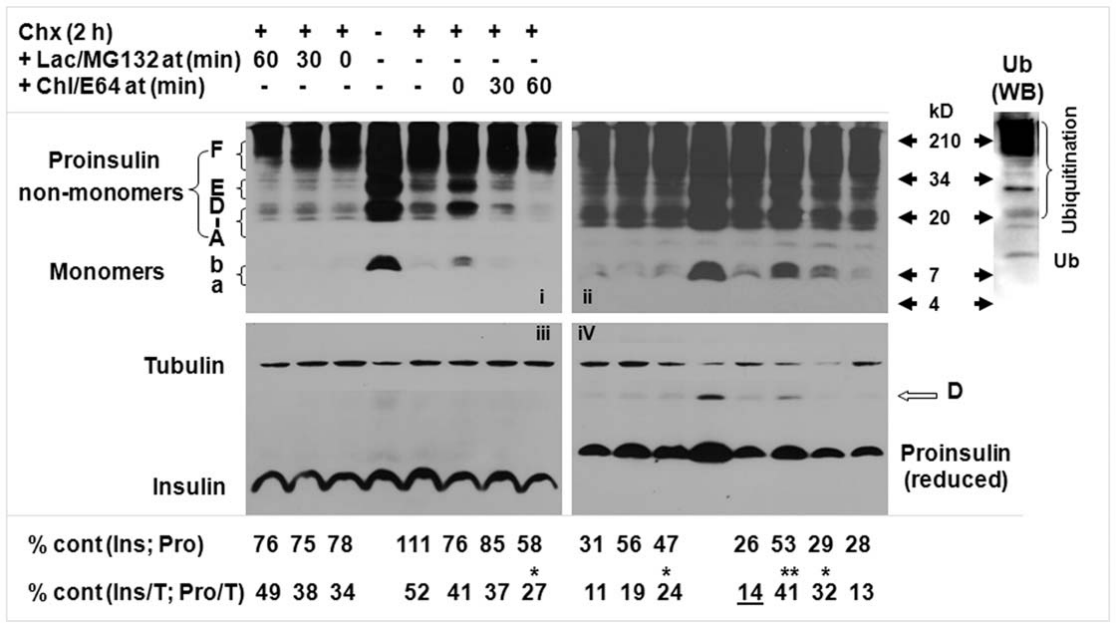
Lysosomal and proteasomal roles at sequential stages of the proinsulin post-translational process. The *Ins2^+/+^* β-cells were cultured under the 5.5 mM glucose concentration for a 24-hour pre-experimental period until treatment. Cycloheximide (Chx; 100 µg/mL); or Chx (100 µg/mL), chloroquine (100 µg/mL), and E-64 (50 µM); or Chx (100 µg/mL), lactacystin (10 µM), and MG-132 (30 µM) was added to the culture media of *Ins2^+/+^* β-cells at 0, 30, or 60 minutes during a 2-hour course with an untreated control. Cellular proteins (30 µg) were separated on16.5% tricine SDS-PAGE under non-reduced/reduced conditions and then examined by immunoblotting. C-peptide (images I and ii, non-reduced condition; image iv, reduced condition); insulin (image iii, non-reduced condition); β-tubulin (image iii, non-reduced condition; image iv, reduced condition); or ubiquitin (right panel, reduced condition; untreated control shown). Images i (shorter exposure), ii (longer exposure), and iii were produced from the same membrane. C-peptide or tubulin immunoblot data shown in image iv were produced from the same membrane. The levels of insulin (Ins) or proinsulin (Pro) compared to the untreated control (% cont [Ins; Pro], non-normalized; % cont [Ins/T; Pro/T], normalized by β-tubulin) are shown at the bottom, below images iii and iv. Ub, ubiquitin; IB, immunoblot. The proinsulin states were denoted previously (1). Statistical significances of the normalized proinsulin level between Chx and other treatments including Chx, or of the normalized insulin level between the chloroquine/E-64 presence from 60 to 120 minutes versus other treatments combining proteasomal/lasosomal inhibitors with Chx were assessed. The data were shown as mean. *, *P*<0.05; **, *P*<0.01; n = 4. The statistical results were detailed in Table S2 and S3.

**Figure 4:**
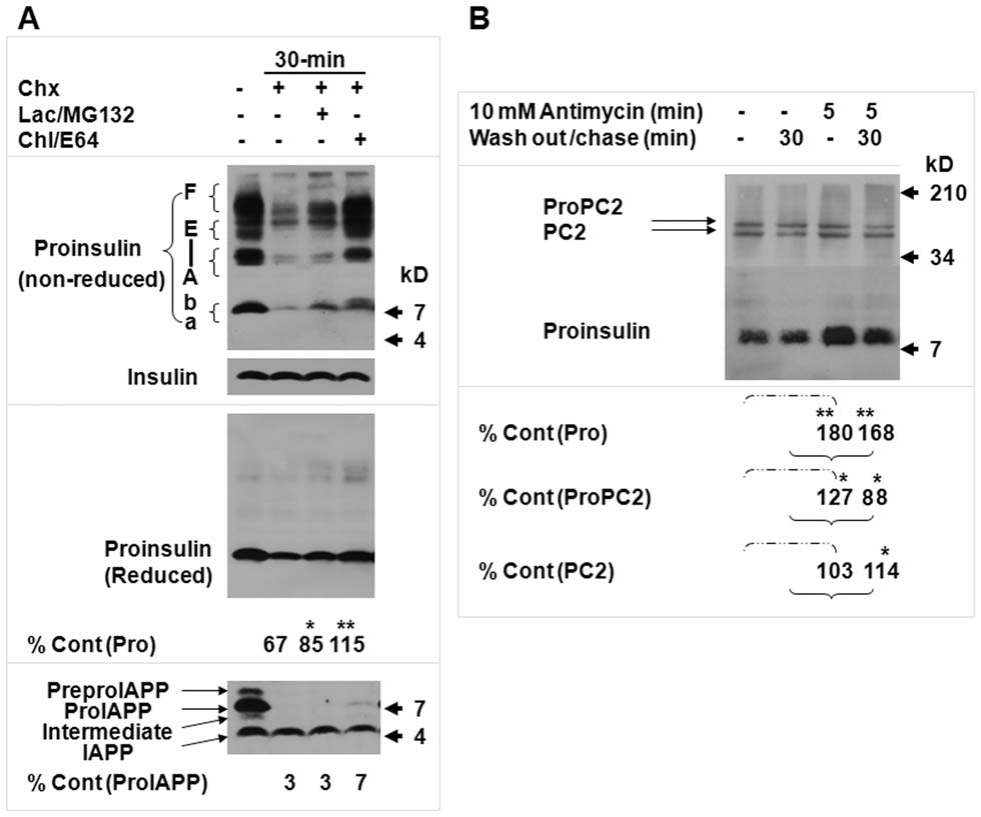
Endogenous proinsulin removal is a substrate-favored process. The *Ins2^+/+^* β-cells were cultured under the 5.5 mM glucose concentration for a 24-hour pre-experimental period until treatment. (A) The *Ins2^+/+^* β-cells were treated with cycloheximide (Chx; 100 µg/mL), Chx (100 µg/mL), lactacystin (10 µM), and MG-132 (30 µM), or Chx (100 µg/mL), chloroquine (100 µg/mL), and E-64 (50 µM) for 30 minutes with an untreated control. Cellular proteins (30 µg) were separated by 16.5% non-reduced/reduced tricine SDS-PAGE and then examined by immunoblotting. The C-peptide or islet amyloid polypeptide (IAPP) immunoblot data under reduced condition in (A) were produced from the same membrane. The percentages of proinsulin (Pro), insulin, or ProIAPP peptide levels in individual treatments compared to the untreated controls are shown at the bottom, below the images in (A). Statistical significances (*P<0.05; **P<0.01) of the proinsulin or proIAPP level between Chx and individual treatments that included Chx were assessed (n = 4). The statistical results were detailed in Table S4. (B) The *Ins2^+/+^* β-cells in 2 dishes were treated with 10 µM of antimycin for 5 minutes with 2 untreated dishes, and then half of the untreated and treated dishes were chased for 30 minutes and the other half harvested immediately. Cellular proteins (30 µg) were separated by 16.5% reduced tricine SDS-PAGE and then examined by C-peptide or PC2 antisera on the same membrane. The percentages of proinsulin (Pro), ProPC2, or PC2 peptide levels in individual treatments compared to the untreated controls are shown at the bottom, below the images in (B). The data were shown as mean. Statistical significances (*P<0.05; **P<0.01) of the proinsulin, proPC2, or PC2 level between the indicated groups were assessed (n = 4). The statistical results were detailed in Table S5.

**Figure 5:**
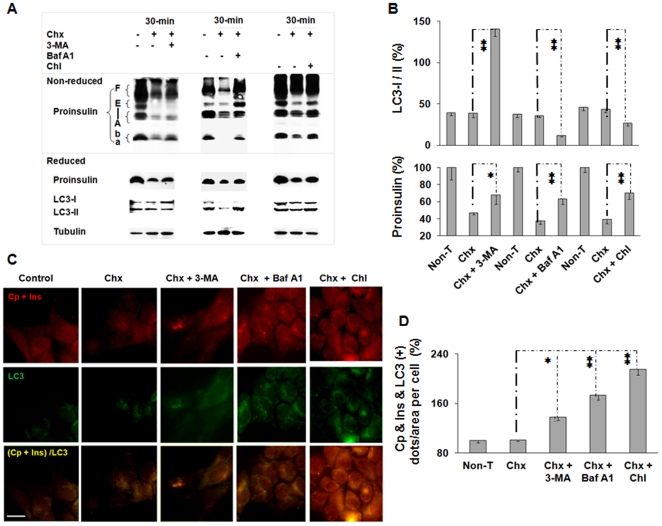
Macroautophagy is a major mechanism in the rapid disposal of insulin precursor in β-cells. The *Ins2^+/+^* β-cells were cultured under the 5.5 mM glucose concentration for a 24-hour pre-experimental period until treatment. (A) The *Ins2^+/+^* β-cells were treated with cycloheximide (Chx; 100 µg/mL), Chx (100 µg/mL) and 3-MA (5 mM), Baf A1 (5 µM), or chloroquine (Chl; 100 µg/mL) for 30 minutes with an untreated control. Cellular proteins (30 µg) were separated by 16.5% non-reduced (upper panels) or reduced (lower panels) tricine SDS-PAGE and then examined by immunoblotting. (B) The upper panel, the immunoreactive LC3-I/II (%) in individual treatments in (A); the lower panel, the percentages of proinsulin levels on reduced gels (that were normalized by tubulin) in individual treatments compared to the untreated controls. (C) The *Ins2^+/+^* β-cells subjected to the same treatments described in (A) were immunostained with antibodies against LC3, C-peptide, and insulin as described in the Materials and Methods. Fluorescent Cy2 (for LC3), Cy3 (for C-peptide and insulin), and their merged images were shown. The scale of bar in (C), 10 µm. (D) The relative levels (%) of the LC3 and (pro)insulin and/or C-peptide positive dots per cell of individual treatments in (C). The data in (B) or (D) were reported as mean ± SD. *P<0.05; **P<0.01, n = 4.

Regardless of addition of other agents to *Ins2^+/+^* β-cells, exposure to Chx (Figures 2, 3, 4), generally enhanced β-tubulin, an internal marker. In the sympathetic neurons, tubulin half-life increased when Chx inhibited protein synthesis [25]. β-tubulin fluctuation in *Ins2^+/+^* β-cells may also be affected by the change in turnover, irrespective of other possibilities, such as an increased proportion of β-tubulin in the whole-cell protein pool as a consequence of imbalanced input and output of abundant secretory protein. Thus, non-normalized accumulations of proinsulin in the addition of Chx and lysosomal or proteasomal inhibitors are generally more severe (Figures 2, 3, 4, 5).

### Roles of lysosomes and proteasomes at sequential stages of the proinsulin post-translational process

Nascent proinsulin normally exits from the ER after 20 minutes [18], [20], moves sequentially into the Golgi and trans-Golgi network, and reaches into the immature insulin granule in about 45 to 60 minutes, and then completes conversion to insulin within the mature granule for release in approximately 2 hours [18]. To determine the roles of lysosomes and proteasomes in the sequential stages, we added selective inhibitors at 0, 30, and 60 minutes in a 2-hour post-translational course with Chx. Compared to the untreated control, 14% of proinsulin molecules were retained in the 2-hour treatment with Chx, 24% with combined Chx/lactocystin/MG-132, and 41% with combined Chx/chloroquine/E-64 (see % below image iv in Figure 3 and details in Table S2 *).* Addition of the combined lysosomal inhibitors thus preserved 27% of proinsulin and addition of the proteasomal inhibitors, 10%, in the 2-hour course (*P*<0.05, n  = 4), an effect resembling that of applying individual inhibitors (Figure 2). Addition from 30 to 120 minutes of chloroquine/E-64 led to the preservation of 18% of proninsulin (*P*<0.05, n  = 4) and addition of lactacystin/MG-132, to a slight (5%) preservation. However, adding either combined inhibitor from 60 to 120 minutes produced no apparent accumulation of proinsulin.

Thus, examination of proinsulin monomers under reduced conditions reveals that proteasomal inhibition within the first 30 minutes of the post-translational 2-hour course indeed preserves 5 to 10% of proinsulin in the islets and cloned β-cells (Figures 1, 2, 3). This slight increase is barely noticeable in the various states of proinsulin shown under non-reduced conditions (Figure 3, images i and ii), but under reduced conditions, the density of the proinsulin monomers and of several bands resistant to dithiothreitol (DTT) and 2-mercaptoethanol and having high molecular weights increased by addition of combined lactocystin/MG-132 or chloroquine/E-64 from 0 to 120 minutes (Figure S2). At least some of these bands, expected to be components of proinsulin states [1], may represent proinsulin modified with non-disulfide covalent bonds by other molecules, such as ubiquitin, because the pattern of proinsulin non-monomer states somewhat resembles that of β-cell protein ubiquitination (Figure 3, image i or ii versus right panel). Therefore, the denoted A to F band/area of proinsulin non-monomer states under non-reduced condition [1] could include aggregates shaped by proinsulin alone and/or with molecular helpers through (non-)covalent interactions. Some aggregates on non-reduced gels or membranes could contain closely migrated DTT-resistant and -sensitive forms.

In untreated cells, the pattern of proinsulin states (Figure 3, images i and ii) is compatible with the pattern observed previously [1]. In all the treatments (with Chx alone or combined with inhibitors), proinsulin monomers (denoted as *a* and *b*) disappeared similarly except when chloroquine with E-64 was added from 0 or 30 minutes to 120 minutes. Monomer *b* , but not *a* , was retained selectively in the 2 excluded treatments; regardless of other possibilities, monomer *a* is the form with native conformation and form *b* , a possible isomer [1]. These observations suggest that the consequent transport and processing (see insulin data below) of the natively folded proinsulin was not markedly altered during the diverse durations of lysosomal or proteasomal inhibition in the limited 2 hours. If prolonged, severe accumulation of non-monomer states and form *b* from lysosomal inhibition from 0 or 30 to 120 minutes could result in consequences, such as insulin production and other biological abnormalities in β-cells.

Immunoblot analysis showed a decrease in normalized insulin level (under non-reduced conditions) in all treatments that included Chx (see image iii in Figure 3 and details in Table S3), which enhanced β-tubulin, as discussed (Figure 2). Insulin levels were roughly similar among the treatments combining Chx with inhibitors. However, a noticeable decrease in insulin in the presence of chloroquine/E-64 from 60 to 120 minutes (Table S3, *p*<0.05, n  = 4) may be linked to the condensation of insulin and stability of the cores in immature granules [20], [26]. It is well known that the insulin secreted in a few hours under low (e.g., 5.5 mM) glucose conditions is only a tiny fraction of the entire cellular insulin content. Thus, except for the excluded treatment, diverse times of lysosomal and proteasomal inhibition during the 2 hours apparently had no obvious effect on insulin content, irrespective of the undetermined secreted insulin. These data further support that the accumulated proinsulin is mainly destined for natural clearance rather than for the customary route in insulin biosynthesis. Interestingly, the immunoreactivity of separated insulin A and/or B chains detected under reduced conditions was noticeably low in the β-cells exposed to chloroquine/E-64 or lactocystin/MG-132 for 2 hours (data not shown), but the reasons are unclear. Nonetheless, in the early post-translational process of proinsulin, a prolonged or permanent alteration or defect caused by any reason(s) appears to have profound toxic consequences on insulin biosynthesis and even β-cell survival; diabetes resulting from proinsulin mutants provides genetic evidence [2]–[4]. In addition, compared to the untreated control, the slightly improved proinsulin conversion to insulin when protein synthesis is inhibited in the treatment of Chx only suggests that a consistent overload may exist for folding in the ER of pancreatic β-cells, which may partially result from the low relative folding rate versus plentiful amounts of insulin precursor made in β-cells [1].

Collectively, these results clearly indicate that the main lysosomal pathway and auxiliary proteasomal pathway function together during the early post-translational process of proinsulin, during which regulations in the ER translocation [15], structural maturation, and (retro-) translocation/transport take place.

### Endogenous proinsulin removal is a substrate-favored process

To determine whether proinsulin is selectively removed among secretory proteins, we conducted immunoblot analysis of the kinetics of insulin and islet amyloid polypeptide (IAPP) precursors in a 30-minute post-translational process. Compared to the untreated control, about 67% of proinsulin molecules were retained with Chx treatment, 85% with Chx/lactacystin/MG-132, and 115% with Chx/chloroquine/E-64; the apparently unaltered insulin content indicated that the customary route of insulin biosynthesis was apparently unaltered in the limited 30 minutes (Figure 4A and Table S4). Thus, approximately 18% of the proinsulin targeted mainly for natural disposal was preserved by 30-minute proteasomal inhibition and 48%, by lysosomal inhibition (Chx versus Chx/lactacystin/MG-132, *P*<0.05; Chx versus Chx/chloroquine/E-64, *P*<0.01; n = 4). The patterns of IAPP and its precursors agreed with those of previous studies [27], [28] in untreated *Ins2^+/+^* β-cells. By contrast, only 3, 3, and 7% of islet amyloid polypeptide precursor (proIAPP) molecules were retained sequentially in the 3 treatments (Table S4 and bottom panel in Figure 4A). Similar to findings in insulin, no significant alteration in IAPP content in all treatments was evident during the 30 minutes chase notwithstanding a slight decline with the chloroquine/E-64 treatment. Theoretically, proIAPP should also undergo ER quality control after removal of signal peptide from preproIAPP by a co/post-translational translocation process (see review: 29) observed in early studies of preproinsulin [30]–[33]. However, lysosomal or proteasomal inhibition did not cause noticeable accumulation of proIAPP in the same course, and although the half-time of proIAPP conversion into IAPP is faster than the half-time of proinsulin conversion into insulin, it still requires about 25 minutes [28]. Obviously, addition of Chx reduces this time considerably, demonstrates by the barely detectable proIAPP in all treatments that include Chx despite the presence of a large amount of proIAPP in the untreated control (Figure 4A). Clearly, the post-translational process of IAPP precursors is enhanced, and the enhancement is barely affected by lysosomal or proteasomal inhibition during the 30 minutes.

Antimycin largely inhibits the heavy clearance of ^35^S-proinsulin in MIN6 β-cells (Figure 1) [1]. In *Ins2^+/+^* β-cells exposed to antimycin for 5 minutes, the level of immunoreactive proinsulin increased to 180%, of prohormone convertase 2 (PC2) precursor to 127%, and of PC2, a functional convertase in insulin biosynthesis, to 103% (Figure 4B and Table S5; *P*<0.01; *P*<0.05; non-significant, n = 4). Antimycin significantly inhibited protein synthesis [34], further supporting that the preferential accumulation of proinsulin results mainly from attenuation of a substrate-favored clearance. After a subsequent 30-minute chase, approximately 168% of proinsulin remained in the β-cells (*P*<0.01, n = 4). In contrast, the slightly accumulated proPC2 disappeared as the PC2 correspondingly increased in the same course (Figure 4B and Table S5). The autoactivation of ProPC2 into PC2 occurs at the trans-Golgi network [18], suggesting that the transport and processing of ProPC2 recovered rapidly during the 30-minute chase. Likewise, prohormone convertase 1/3 (PC1/3), another convertase autoactivated in the ER [18], does not undertake noticeable clearance when proinsulin is heavily removed [1]. Thus, endogenous proinsulin is selectively and largely removed by energy-sensitive clearance processes during the maturation and transport of secretory proteins.

### A role of macroautophagy in the removal of endogenous proinsulin

To establish whether the role of lysosomes in proinsulin disposal is mainly achieved through macroautophagy and/or other mechanisms like CMA, we next examined the effect of autophagy inhibitors 3-methyladenine (3-MA) and bafilomycin A1 (Baf A1) versus chloroquine on kinetics of proinsulin. Compared to chloroquine that targets the entire lysosomal lumen, 3-MA inhibits macroautophagy by blocking autophagosome formation and Baf A1 prevents maturation of autophagic vacuoles by inhibiting fusion between autophagosomes and lysosomes and inhibiting lysosomal acidification [35], [36]. In a 30 minutes post-translational course by presence of Chx in *Ins2^+/+^* β-cells, addition of 3-MA (5 mM), Baf A1 (5 µM), or chloroquine (100 µg/mL) resulted in changes in the ratio of microtubule-associated protein 1 LC3-I/II, an internal marker showing activities of the autophagic process. LC3-I is a cytoplasmic form. In contrast, LC3-II, a product converted from LC3-I, is an autophagic membrane binding form and its amount correlates well with the number of autophagosomes [37]. The occurrence of the increased LC3-II and LC3-I/II ratio by Baf A1 or chloroquine and the inhibition of LC3-I conversion to LC3-II by 3-MA (Figure 5A and B) is in an agreement with previous observations in pancreatic β-cells and other cell types [37], [38]. Optimal concentrations of Baf A1 in our acute post-translational studies (data not shown) were higher than the concentrations used in the studies reported previously [36], [38]. These results indicate the autophagic process blocked by these agents.

In the same post-translational processing course, approximately 20, 29, and 37% of the proinsulin targeted mainly for disposal was preserved by the presence of 3-MA, Baf A1, or chloroqunine, respectively (Figure 5A and B *; P*<0.05; *P*<0.01; *P*<0.01; n = 4), The results indicate these inhibitors that affect sites diversely in the macroautophagic process or lysosomal compartments preserve different amounts of proinsulin, and suggests macroautophagy is an important mechanism for rapid proinsulin disposal. In consistent with this observation, immunohistochemistry examinations exposed a similar tendency in the increase of LC3 and (pro)insulin and/or C-peptide co-localized dots induced by these three agents compared to the control treatment with Chx only in a 30 minutes post-translational course (Figure 5C and D *; P*<0.05; *P*<0.01; *P*<0.01; n = 4). These results clearly show that macroautophagy is an important but not the sole mechanism for the role of lysosomes in the insulin precursor disposal of β-cells. This is because the amount of proinsulin preserved by 3-MA (that blocks autophagosome formation) is less than that by Baf A1, which prevents fusion between autophagosomes and lysosomes and affect acidification of lysosome via the inhibition of vacuolar-type H^+^-ATPase. Moreover, the most plentiful amount of proinsulin is preserved by chloroquine, which is a powerful inhibitor of lysosomal acidification via preferential accumulations in the lysosomal lumen of cells [21], [22].

## Discussion

### Roles of the lysosomal and proteasomal pathways in the early post-translational processing of insulin precursor

Over 30% of ^35^S-Met-labeled nascent proinsulin in islets or 30 to 60% in MIN6 β-cells is rapidly removed within a 20-minute chase (Figure 1), a finding consistent with previous observations [1]. Immunoblotting analysis further validated that at least 30% of total proinsulin in *Ins2^+/+^* β-cells is cleaned, mainly during a 60-minute post-translational process (Figures 2, 3, 4). Thus, a large fraction of proinsulin is unquestionably removed during the natural process of maturation and transport despite the degree of removal seems to be somewhat different between primary and cloned β-cells. In this study with selective inhibitors, we define the lysosomal and proteasomal pathways as involved in the proinsulin removal. Both metabolic-labeling and immunoblotting studies suggest that lysosomal autophagy is primarily responsible for the rapid proinsulin disposal following translation (Figures 1, 2, 3, 4), and both pathways carry out the subsequent basal clearance, which possibly occurs after translocation into the ER. Thus, multiple mechanisms at the molecular level yet to be defined participate in PIHO at the early secretory pathway. These findings also provide a better understanding of the cellular mechanisms underlying (a) the occurred β-cell dysfunction and diabetes in the mice with deletion of Atg7 (which is an essential molecule in the formation of autophagosomes) [39], [40] and (b) a showed link of the proteasomal pathway and insulin secretion [41]–[43]. The proinsulin non-monomer states and their dual fates that were recently exposed [1] are consistently observed in this study (Figures 1, 2, 3, 4). The data further enforce the *in vivo* formation nature of these non-monomer states [1]. Molecular helpers located in the non-monomer states, which may facilitate regulations of proinsulin maturation and disposal [1], remain to be identified.

Of note, applications of (a) C-peptide antisera, (b) C-peptide immunoblotting and metabolic-labeling approaches for analysis of proinsulin states/levels, and (c) selective inhibitors are important in disclosing the roles of lysosomal and proteasomal pathways in normal proinsulin PHIC. As shown, proinsulin non-monomers (and even monomer *b*) showed lower affinity with the high conformation-dependent insulin antisera [44] than with C-peptide antisera (see proinsulin states on a membrane detected by insulin or C-peptide antisera, Figures 3, images i-iii, and S3). The finding from these immunoblot results is consistent with that found recently in immunoprecipitation studies [1]. These data further suggest application of C-peptide antisera is helpful for adequate detection of incompletely folded proinsulin non-monomers, although the insulin antisera are employed as master antibodies in the early and current study of proinsulin folding and related β-cell biology.

On the other hand, traditional pulse-chase approached showed that without C-peptide immunoblotting, proinsulin state analysis, and application of selective inhibitors, the states and rapid disposal of proinsulin cannot adequately be determined. Moreover, only studies with electron microscope disclosed lysosomal degradation of proinsulin, and those with some difficulty, because previous studies within the crinophagic body, a compartment fused with the lysosome for turnover of insulin granules, barely detected C-peptide immunoreactivities [45]. A possible explanation is that soluble C-peptide degrades more easily/rapidly than the relatively insoluble insulin crystals in the crinophagic body [45].

### Preferential disposal of insulin precursor in the maturation and transport process of secretory proteins in β-cells

Our immunoblot analysis of insulin, IAPP, and PC2 proteins and their precursors clearly indicates the preferential disposal of endogenous proinsulin among secretory proteins during their maturation and transport processes in β-cells (Figure 4). This observation is in agreement with the previous finding in metabolic-labeling studies that a large fraction of nascent proinsulin is selectively removed by comparison with other secretory proteins such as IAPP, PC1/3, and their precursors [1]. Such a selective disposal may be linked to the intrinsic properties of proinsulin *in vivo*, such as its low relative folding rate and proneness to aggregation [1]. These properties could depend mainly on the topology of (pre)proinsulin determined by the primary amino acid sequence [46]. In addition, the mechanisms of substrate-specific translocation/ERAD for some proteins (see reviews: 5, 6) may be similarly implicated in the favored disposal of endogenous proinsulin.

### Mechanisms for the removal of endogenous proinsulin in β-cells

The results (Figures 1, 2, 3, 4) in this study at the first time suggest that multiple disposal mechanisms participate in proinsulin disposal for the normal maintenance of PIHO at the early secretory pathway. Further analysis with (non)selective macroautophogy inhibitors and marker clearly defined that macroautophagy is an important mechanism involved (Figure 5). In addition to the defined role of macroautophagy in the bulky and rapid removal of proinsulin, CMA may be a likely participating mechanism considering the substrate-favored natures of insulin precursor disposal. Likewise, the occurrence of proinsulin disposal extends beyond a 30 minutes post-translational course (Figure 3), suggesting that the customary Golgi–endosome–lysosome path may be also active for proinsulin disposal during the natural maturation and transport processing for insulin production. This is in agreement with the observations in early insulin biosynthesis studies [18]. Moreover, our data (Figures 1, 2, 3, 4) suggest that the ERAD proteasomal pathway also plays a role in counterbalancing the natural proinsulin maturation and transport processing under physiological glucose concentrations.

The sustained active rapid and favored clearance route of insulin precursor may serve a protective or adaptive mechanism for maintenance of the ER function in addition to its participating in the early control of insulin production. This route can early eliminate excessive or misfolded insulin precursor when overloads occur by stimuli (such as high glucose) or proinsulin folding processing is disturbed by various influences. Nonetheless, a prolonged or permanent alteration/defect in the disposal and/or counterbalanced maturation routes could result in profound toxic consequences on insulin biosynthesis and whole function of β-cells. For example, heterogeneous mutations at the residues beyond the cleavage sites in the preproinsulin molecule produce hyper- and hypo(pro)insulinemia, contrasting syndromes in mouse and/or humans [2]–[4], [18]. The contrasting syndromes would be a result of atypical maturation and disposal process integrations primarily/largely induced by varied mutants.

In summary, our improved metabolic-labeling and C-peptide immunoblotting approaches revealed the favored and bulky removal of insulin precursor at the early stage of normal insulin biosynthesis under the conditions with mimic physiological glucose concentrations. This study determined the roles of lysosomal and proteasomal pathways in the normal maintenance of PIHO, and defined the physiological participation of lysosomal autophagy in the regular quality control of secretory proteins. The findings will help elucidate the actual pathological defects and mechanisms in diabetes with PIHO disorders. Whether the ubiquitin-proteasome and autophagy-lysosome systems are cross-talked or independent in (a) typical proinsulin disposals and what the real underlying molecular mechanisms are deserve further study. Knowing the answers to these questions might ultimately lead to novel means to preserve or restore insulin production capacity in prevention and treatment of diabetes.

## Materials and Methods

### Ethics statement

All animal and tissue sample experiments have been approved by the Institutional Animal Care and Use Committee (IACUC) of The Ohio State University (protocol number 2007A0040 and 2010A0024) and were performed in accordance with the guidelines of the National Institutes of Health and The Ohio State University.

### Materials

In this study, we applied antibodies against mouse C-peptide II (made in the lab with help from Proteintech Group, Inc., Chicago, IL, USA), rat C-peptide II (Linco Research Inc., St. Louis, MO, USA), insulin (Dako North America, Inc., Carpinteria, CA, USA), and tubulin (Sigma-Aldrich, St. Louis, Mi, USA), ubiqutin (Sc-8017; Santa Cruz Biotechnology, Inc., Santa Cruz, CA, USA), LC3 (NB100-2331; Novus Biologicals Inc., Littleton, CO), PC2 (kindly provided by Dr. Donald F. Steiner, Chicago), and IAPP antibodies (kindly provided by Dr. P. Westermark, Linköping, Sweden). We obtained N-ethylmaleimide (NEM), Chx, antimycin, brefeldin A, bafilomycin A1, chloroquine, 3-methyladenine, E-64, and MG-132 from Sigma; lactacystin from Cayman Chemical Company, Ann Arbor, MI, USA; Met-free Dulbecco's modified eagle's medium (DMEM) and Roswell Park Memorial Institute (RPMI) 1640 from Invitrogen (Carlsbad, CA, USA); and protease inhibitor cocktail from Roche Applied Science (Indianapolis, IN, USA). We obtained Immobilon-P^SQ^ membrane from Millipore (Bedford, MA, USA); [^35^S]-Met from Perkin Elmer (Waltham, MA, USA), and C57BL/6J mice from The Jackson Laboratory (Bar Harbor, ME, USA). The cloned *Ins2^+/+^* β-cells were kindly provided by Dr. H. Kubota (in the laboratory of Dr. K. Nagata, Kyoto, Japan), and MIN6 β-cells were a gift from Dr. Donald F. Steiner (Chicago, IL, USA).

### Islet preparation and cell culture

In this study, all operations and materials used were kept away from reducing reagents unless specifically stated. Islet isolation and culture of islets and cell lines were described previously [2], [24]. Briefly, we incubated the cells in 35-mm dishes in DMEM (25.5 mM glucose) supplemented with 10% fetal bovine serum plus (FBS) at 37°C with 5% CO_2_/95% O_2_ until 80–90% confluence for 24-hour pre-experimental and various experimental treatments with 5.5 mM glucose. We cultured isolated islets overnight in 10% FBS/RPMI 1640 (11 mM glucose) until treatment.

### Pulse and/or chase

After 30 minutes of preincubation in Met-free DMEM (for MIN6 β-cells) or Met-free RPMI 1640 medium (for islets), mouse islets or MIN6 β-cells were labeled with ^35^S-Met in the same media. We utilized pre-balanced complete DMEM/10% FBS (5.5 mM glucose) or RPMI 1640/10% FBS (11 mM glucose) media with and without various inhibitors in the various chase tests. After pulse and/or chase incubations, islets and MIN6 β-cells were quickly washed with PBS containing 20-mM NEM and immediately lysed in the immunoprecipitation buffer and frozen at −80°C. After the various post-translational courses (Figures 2, 3, 4, 5), the *Ins2^+/+^* β-cells were quickly washed and immediately lysed in the tricine gel sample and frozen at −80°C.

### Protein extraction buffers and sample preparations

We extracted cellular proteins by the tricine gel sample buffer (100 mM Tris, pH 6.8, 1% sodium dodecyl sulfate [SDS], 20% glycerol, 20-mM N-ethylmaleimide, and 0.02% Coomassie Blue) for immunoblotting and by immunoprecipitation buffer (50 µM Tris.HCl, pH 7.4, 100 mM NaCl, 2.5 mg/mL BSA, 1% Triton X-100, 20 mM NEM, and protease inhibitor cocktail) for immunoprecipitation analysis.

Lysed cells were then subjected to sample preparations described previously [1].

### SDS-PAGE, immunoblotting, immunoprecipitation, and radioautography

We boiled proteins for 10 minutes in tricine gel sample buffer with and without 280 mM 2-mercaptoethanol for separation on the tricine SDS-PAGE without urea (10% T and 5% C, Figure 1B, C; 16.5% T and 5% C, Figures 2, 3, 4, 5; T denotes the total percentage concentration of both acrylamide and bisacylamide; C denotes the percentage concentration of the bisacylamide relative to the total concentration). Immunoblotting and immunoprecipitation were performed following procedures described previously [1]. For autoradiography, we dried membrane and fixed gels with labeled materials for exposure on x-films.

### Immunohistochemistry

*Ins2^+/+^* β-cells seeded on cover-glass were cultured in the customary 10% FCS/DMEM medium for 48 h and then subjected to 24-hour pre-experimental and 30 minutes experimental treatments with 5.5 mM glucose. After treatments, *Ins2^+/+^* β-cells were fixed with pre-cold methanol for 5 minutes and then immunostained with antibodies against LC3 (1∶150), C-peptide (1∶200), and insulin (1∶500) as described previously [2]. Fluorescent images of Cy2 for LC3 and Cy3 for C-peptide and insulin were examined with an Axiovert 200 microscope (Carl Zeiss, Oberkochen, Germany). To quantify the LC3 and (pro)insulin and/or C-peptide co-localized dots per cell, the number/area of the co-localized dots in 15 cells of individual treatments were counted from four independent experiments.

### Quantitative analysis of immunoreactivity and radioactivity

To quantify the density and radioactivity of proteins (area and/or gel slices), we used National Institutes of Health (NIH) ImageJ software and/or liquid scintillation counter and gamma counter (Beckman Coulter, Inc., Brea, CA, USA).

### Data Analysis

Data are presented as the mean or mean ± standard deviation (SD, n = 3 or 4). We assessed statistical significance (*P<0.05; **P<0.01) by two-tailed Student’s t-test.

## Supporting Information

Figure S1 **No proinsulin was detected in the immunoprecipitates of control sera.** After 24-hour pre-experimental culture with the 5.5 mM glucose concentration, MIN6 β-cells were chased for 3 minutes after a 5 minutes pulse with ^35^S-Met. Cellular proteins were then subjected to immunoprecipitation with insulin (Ins) and C-peptide (Cp) antisera or with control sera (C). Equal amounts of individual immunoprecipitates were resolved by 10% tricine non-reduced and/or reduced SDS-PAGE for radiography.
(TIF)

Figure S2 **A longer exposure image of cellular proinsulin in the C-peptide immunoblot analysis after resolution under reduced condition.** Figure 3, image iv has a shorter exposure. This longer exposure image showed that the density of several dithiothreitol (DTT)-resistant bands of high molecular weight increased by addition of Chx, lactocystin, and MG-132 or Chx, chloroquine, and E-64 from 0 to 120 minutes compared to the addition of Chx alone in the same course.
(TIF)

Figure S3 **A longer exposure image of cellular (pro)insulin in the insulin immunoblot analysis after resolution under non-reduced condition.** On the same membrane, the various proinsulin non-monomer states that were clearly detected by C-peptide antisera (see Figure 3, images i and ii) were weakly detected by conformation-dependent insulin antisera at shorter (Figure 3, image iii) and longer (this image) exposure conditions.
(TIF)

Table S1 **Relative levels of labeled proinsulin monomers in the individual treatments shown on the reduced gel in** **Figure 1B** **.**
(PDF)

Table S2 **Relative levels of proinsulin monomers in the individual treatments shown in the image IV of** **Figure 3** **.**
(PDF)

Table S3 **Relative levels of insulin monomers in the individual treatments shown in the image III of** **Figure 3** **.**
(PDF)

Table S4 **Relative levels of proinsulin or ProIAPP monomers in the individual treatments shown on the reduce gel in** **Figure 4A** **.**
(PDF)

Table S5 **Relative levels of proinsulin, ProPC2, or PC2 proteins in the individual treatments shown on the reduce gel in** **Figure 4B** **.**
(PDF)
