# Supplementary figures and images for: Substrate-Favored Lysosomal and Proteasomal Pathways Participate in the Normal Balance Control of Insulin Precursor Maturation and Disposal in β-Cells
Source: PLoS One. 2011 Nov 10;6(11):e27647. doi: 10.1371/journal.pone.0027647 (PMC3213186; doi:10.1371/journal.pone.0027647)

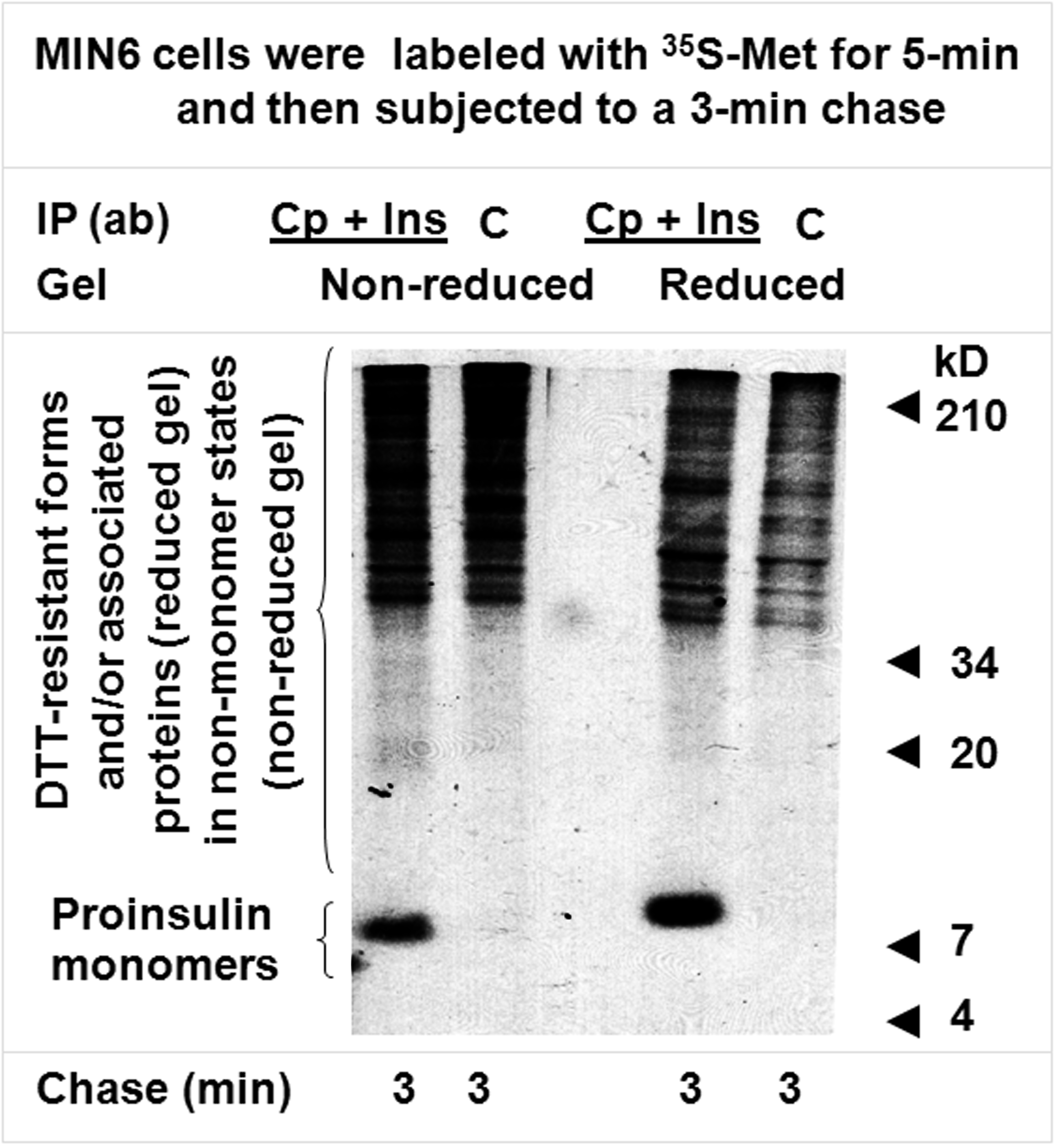

Supplement: Figure S1 — No proinsulin was detected in the immunoprecipitates of control sera. After 24-hour pre-experimental culture with the 5.5 mM glucose concentration, MIN6 β-cells were chased for 3 minutes after a 5 minutes pulse with 35S-Met. Cellular proteins were then subjected to immunoprecipitation with insulin (Ins) and C-peptide (Cp) antisera or with control sera (C). Equal amounts of individual immunoprecipitates were resolved by 10% tricine non-reduced and/or reduced SDS-PAGE for radiography. (TIF) [file pone.0027647.s001.tif]

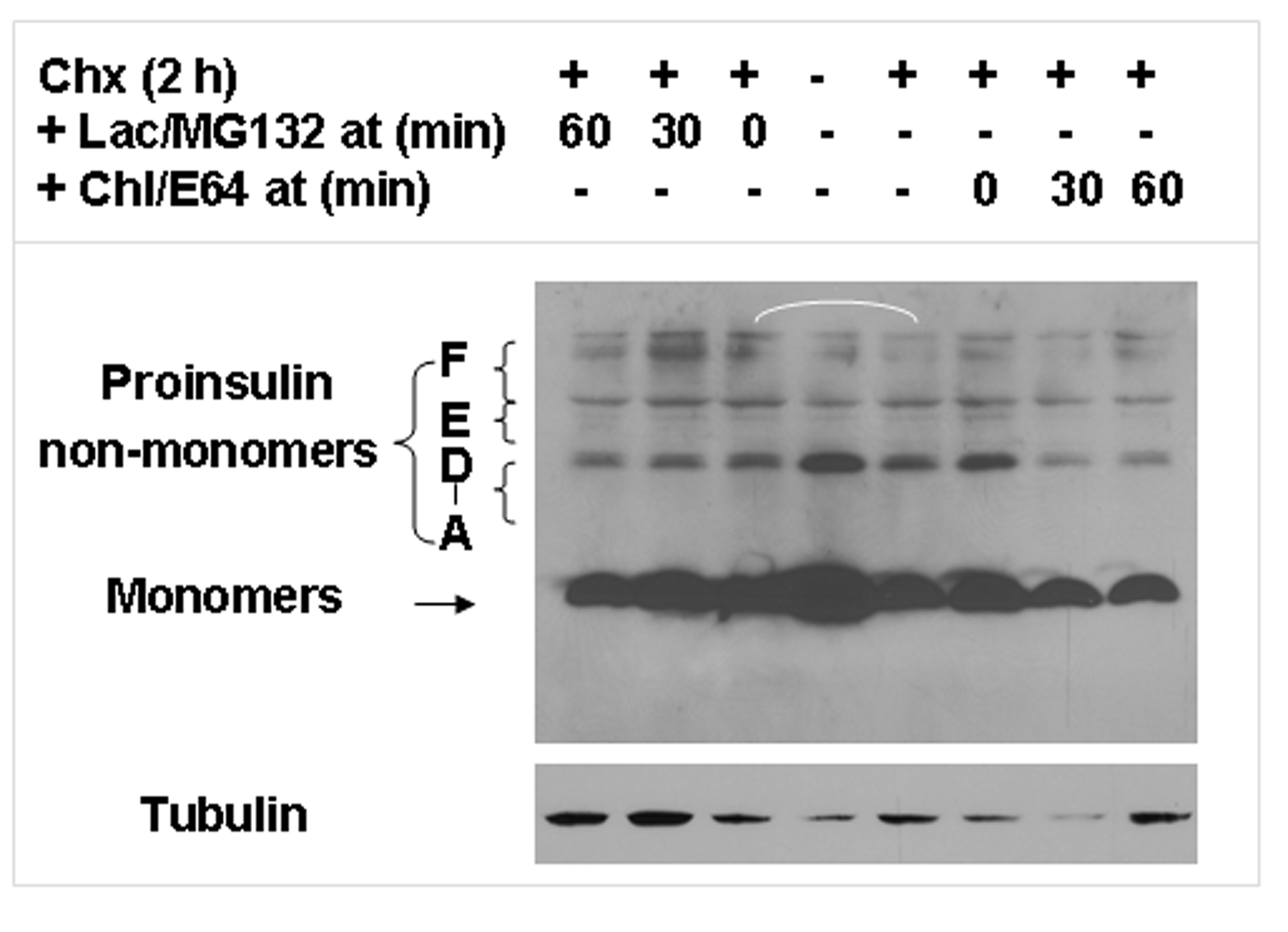

Supplement: Figure S2 — A longer exposure image of cellular proinsulin in the C-peptide immunoblot analysis after resolution under reduced condition. Figure 3, image iv has a shorter exposure. This longer exposure image showed that the density of several dithiothreitol (DTT)-resistant bands of high molecular weight increased by addition of Chx, lactocystin, and MG-132 or Chx, chloroquine, and E-64 from 0 to 120 minutes compared to the addition of Chx alone in the same course. (TIF) [file pone.0027647.s002.tif]

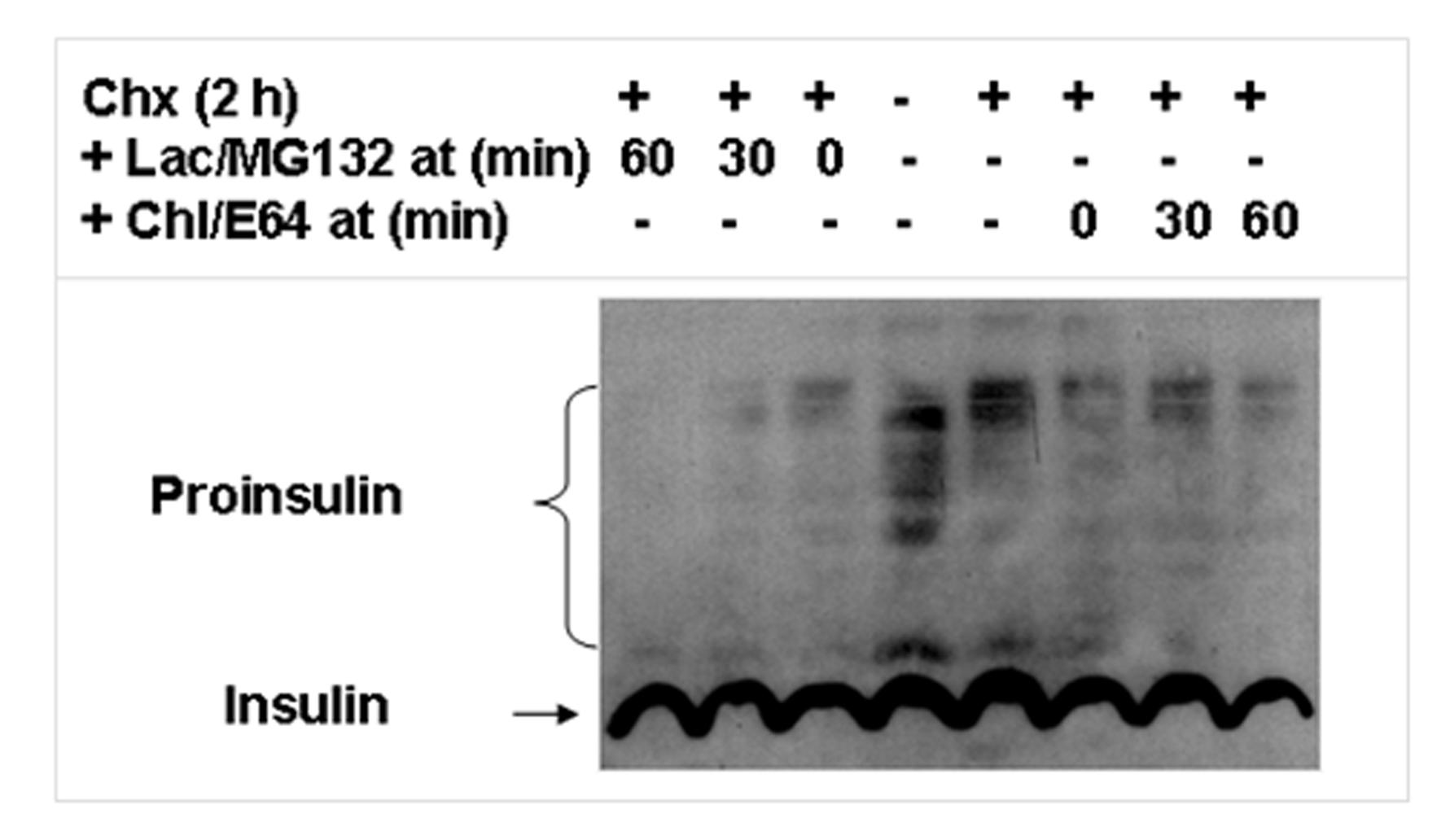

Supplement: Figure S3 — A longer exposure image of cellular (pro)insulin in the insulin immunoblot analysis after resolution under non-reduced condition. On the same membrane, the various proinsulin non-monomer states that were clearly detected by C-peptide antisera (see Figure 3, images i and ii) were weakly detected by conformation-dependent insulin antisera at shorter (Figure 3, image iii) and longer (this image) exposure conditions. (TIF) [file pone.0027647.s003.tif]
