# Supplementary material for: Substrate-Favored Lysosomal and Proteasomal Pathways Participate in the Normal Balance Control of Insulin Precursor Maturation and Disposal in β-Cells
Source: PLoS One. 2011 Nov 10;6(11):e27647. doi: 10.1371/journal.pone.0027647 (PMC3213186; doi:10.1371/journal.pone.0027647)
Supplement: Table S1 — Relative levels of labeled proinsulin monomers in the individual treatments shown on the reduced gel in Figure 1B . (PDF) [file pone.0027647.s004.pdf]

Table S1. Relative levels of labeled proinsulin monomers in the individual treatments shown on the reduced gel (Figure 1B)

Monomers	C3	C15	C15Chl	C15Lac	C15BFA	C15Antimycin
Percentage	51.1	39.3	86.1	48.5	90.8	100
SD	2.8	2.4	5.9	3.4	6.5	6.9
P (C15 vs. others)	<0.005		<0.005	<0.005	<0.005	<0.005

C3, 3-min chase; C15, 15-min chase; C15Chl, 15-min chase with chloroquine; C15Lac,15-min chase with lactacystin; C15BFA, 15-min chase with brefeldin A; C15Antimycin, 15-min chase with antimycin.
