# Supplementary material for: Substrate-Favored Lysosomal and Proteasomal Pathways Participate in the Normal Balance Control of Insulin Precursor Maturation and Disposal in β-Cells
Source: PLoS One. 2011 Nov 10;6(11):e27647. doi: 10.1371/journal.pone.0027647 (PMC3213186; doi:10.1371/journal.pone.0027647)
Supplement: Table S2 — Relative levels of proinsulin monomers in the individual treatments shown in the image IV of Figure 3 . (PDF) [file pone.0027647.s005.pdf]

Table S2. Relative levels of proinsulin monomers in the individual treatments shown in the image iv of Figure 3

	Chx	-	2 h	2 h	2 h	2 h	2 h	2 h	2 h
Add at (min)	Chl/E64	-	-	0	30	60	-	-	-
Add at (min)	Lac/ MG-132	-	-	-	-	-	0	30	60
Mean (%)		100.0	14.2	41.3.	32.2	13.4	24.4	18.7	11.3
SD		4.3	4.1	5.3	5.1	4.2	4.9	4.7	3.2
P				< 0.01	< 0.05	ns	< 0.05	ns	ns
n		4	4	4	4	4	4	4	4

The *Ins2*$^{+/+}$ β-cells were cultured under the 5.5 mM glucose concentration for a 24-hour pre-experimental period until treatment. Cycloheximide (Chx; 100 µg/mL); or Chx (100 µg/mL), chloroquine (Chl; 100 µg/mL), and E-64 (50 µM); or Chx (100 µg/mL), lactacystin (10 µM), and MG-132 (30 µM) was added to the culture media of *Ins2*$^{+/+}$ β-cells at 0, 30, or 60 minutes during a 2-hour course with an untreated control. Cellular proteins (30 µg) were separated on16.5% tricine SDS-PAGE under non-reduced/reduced conditions and then examined by immunoblotting. Here shows the relative levels of proinsulin monomers in the individual treatments in the image iv of Figure 3. Mean (%): percentage of the (average) proinsulin level in individual treatments compared to the untreated control and normalized by β-tubulin (shown below image iv in Figure 3). *P,* two tailed *t*-test (Chx versus other individual treatments). ns, non-significant.
