# Supplementary material for: Substrate-Favored Lysosomal and Proteasomal Pathways Participate in the Normal Balance Control of Insulin Precursor Maturation and Disposal in β-Cells
Source: PLoS One. 2011 Nov 10;6(11):e27647. doi: 10.1371/journal.pone.0027647 (PMC3213186; doi:10.1371/journal.pone.0027647)
Supplement: Table S4 — Relative levels of proinsulin or ProIAPP monomers in the individual treatments shown on the reduce gel in Figure 4A . (PDF) [file pone.0027647.s007.pdf]

Table S4. Relative levels of proinsulin or ProIAPP monomers in the individual treatments shown on the reduce gel in Figure 4A

	Chx	-	30 min	30 min	30 min
	Lac + MG-132	-	-	+	-
	Chl + E64	-	-	-	+
Proinsulin mean (%)		100.0	66.9	85.2	115.3
SD		4.9	5.1	5.7	6.1
P				< 0.05	< 0.01
ProIAPP mean (%)		100.0	3.2	3.3.	7.4
SD		4.8	1.1	1.8	3.1
P				ns	ns
n		4	4	4	4

The *Ins2*$^{+/+}$ β-cells were cultured under the 5.5 mM glucose concentration for a 24-hour pre-experimental period until treatment. Cycloheximide (Chx; 100 µg/mL); or Chx (100 µg/mL), chloroquine (100 µg/mL), and E-64 (50 µM); or Chx (100 µg/mL), lactacystin (10 µM), and MG-132 (30 µM) was added to the culture media of *Ins2*$^{+/+}$ for 30 minutes with an untreated control. Cellular proteins (30 µg) were separated by 16.5% non-reduced/reduced tricine SDS-PAGE and then examined by immunoblotting. The C-peptide or islet amyloid polypeptide (IAPP) immunoblot data under reduced condition were produced from the same membrane. Here shows the relative levels of proinsulin or ProIAPP monomers in the individual treatments shown on the reduce gel in Figure 4A. Mean (%): percentage of the (average) proinsulin or proIAPP level in individual treatments compared to the untreated control. *P,* two tailed *t*-test (Chx versus other individual treatments). ns, non-significant.
