# Supplementary material for: Substrate-Favored Lysosomal and Proteasomal Pathways Participate in the Normal Balance Control of Insulin Precursor Maturation and Disposal in β-Cells
Source: PLoS One. 2011 Nov 10;6(11):e27647. doi: 10.1371/journal.pone.0027647 (PMC3213186; doi:10.1371/journal.pone.0027647)
Supplement: Table S5 — Relative levels of proinsulin, ProPC2, or PC2 proteins in the individual treatments shown on the reduce gel in Figure 4B . (PDF) [file pone.0027647.s008.pdf]

Table S5. Relative levels of proinsulin, ProPC2, or PC2 proteins in the individual treatments shown on the reduce gel in Figure 4B

	Antimycin	-	-	5 min	5 min
	Change media	-	+	-	+
	Chase	0 min	30 min	0 min	30 min
Proinsulin mean (%)		100.0	100.0	180.1	168.3
SD		4.6	5.4	7.7	6.9
P				< 0.01	< 0.01
ProPC2 mean (%)		100.0	100.0	127.2	88.3
SD		5.1	4.6	5.8	4.1
P				< 0.05	< 0.05
PC2 mean (%)		100.0	100.0	103.0	114.2
SD		4.4	4.7	4.8	4.9
P				ns	< 0.05
n		4	4	4	4

The *Ins2*$^{+/+}$ β-cells were cultured under the 5.5 mM glucose concentration for a 24-hour pre-experimental period until treatment. Cycloheximide (Chx; 100 µg/mL); or Chx (100 µg/mL), chloroquine (100 µg/mL), and E-64 (50 µM); or Chx (100 µg/mL), lactacystin (10 µM), and MG-132 (30 µM) was added to the culture media of *Ins2*$^{+/+}$ for 30 minutes with an untreated control. Cellular proteins (30 µg) were separated by 16.5% non-reduced/reduced tricine SDS-PAGE and then examined by immunoblotting. The C-peptide or islet amyloid polypeptide (IAPP) immunoblot data under reduced condition were produced from the same membrane. Here shows the relative levels of proinsulin or ProIAPP monomers in the individual treatments shown on the reduce gel in Figure 4A. Mean (%): percentage of the (average) proinsulin or proIAPP level in individual treatments compared to the untreated control. *P,* two tailed *t*-test (Chx versus other individual treatments). ns, non-significant.
